# Women's body dissatisfaction, physical appearance comparisons, and Instagram use throughout the COVID‐19 pandemic: A longitudinal study

**DOI:** 10.1002/eat.23827

**Published:** 2022-10-21

**Authors:** Helena Vall‐Roqué, Ana Andrés, Himar González‐Pacheco, Carmina Saldaña

**Affiliations:** ^1^ Department of Clinical Psychology and Psychobiology, Faculty of Psychology Universitat de Barcelona Barcelona Spain; ^2^ Faculty of Psychology, Education Sciences and Sport Ramon Llull University Barcelona Spain; ^3^ Canary Islands Health Research Institute Foundation (FIISC) Tenerife Spain; ^4^ Institut de Neurociències, Universitat de Barcelona Barcelona Spain

**Keywords:** appearance comparison, body dissatisfaction, body image, COVID‐19, eating disorders, Instagram, longitudinal, social networks

## Abstract

**Objective:**

This study aimed to determine the evolution of Instagram use, body dissatisfaction and physical appearance comparisons throughout the coronavirus disease‐2019 (COVID‐19) pandemic, and to explore whether there was a relationship between the changes in Instagram use throughout the pandemic and body dissatisfaction and physical appearance comparisons.

**Method:**

A total of 272 Spanish women (16–70 years old) were followed‐up across four waves of assessment between November 2019 (before the pandemic started) and July 2021. Body dissatisfaction, social appearance comparisons, and Instagram use were assessed using the Eating Disorders Inventory‐3, the Physical Appearance Comparison Scale‐Revised, and an ad hoc questionnaire for Instagram use, respectively.

**Results:**

No statistically significant changes were found in the frequency of Instagram use, nor on the proportion of women following appearance‐focused accounts on Instagram, among the data collection periods. Body dissatisfaction significantly increased from T1 to T4, and physical appearance comparisons significantly increased from T1 to T2, T3, and T4. These increases were not found to be significant for those with eating disorder risk. No significant differences were found in body dissatisfaction and physical appearance comparisons depending on whether participants' frequency of Instagram use had changed or remained the same, or whether they had started/stopped/continued following appearance‐focused accounts on Instagram during the pandemic.

**Discussion:**

Women's body dissatisfaction and physical appearance comparisons seem to have increased throughout the pandemic. The experiences of individuals with eating disorder risk throughout the pandemic, and the relationship between the pandemic and Instagram use, might be complex and need further research.

**Public Significance:**

This study suggests that women's body dissatisfaction and physical appearance comparisons have increased throughout the COVID‐19 pandemic. However, this increase might not be as clear for those who had eating disorder risk before the pandemic. Instagram frequency of use, and the percentage of women following appearance‐focused accounts on Instagram, do not seem to have significantly increased. More research is needed to explore the impact of the pandemic.

## 1 INTRODUCTION

The emergence of the highly infectious coronavirus disease‐2019 (COVID‐19) has posed a significant threat to global health. The rapid spread of the virus across the world resulted in several governments implementing drastic health measures, including lockdowns, physical distancing, closure of nonessential services, and travel restrictions (Castex et al., 2021).

In Spain, on March 14, 2020, a state of emergency was declared, placing the entire country in lockdown. All nonessential work activity was suspended, and the population was mandated to remain at home. After the initial 6 weeks, restrictions were gradually lifted. However, some health and social measures lasted for more than 1 year or are still in place at the time of writing this article (e.g., wearing a face mask in some indoor spaces and public transport). Six waves of coronavirus infections have been reported in Spain since the beginning of the pandemic until May 2022.

Since the outbreak of the pandemic, several researchers and clinicians have expressed concern about its impact on mental health (World Health Organization Regional Office for Europe, 2022). It has been suggested that the pandemic increased the prevalence of mental health problems such as depression, anxiety, distress, and insomnia (Jin et al., 2021; Wu et al., 2021).

Eating habits, appearance concerns, eating disorder risk, and disordered eating might have also worsened with the pandemic (e.g., Linardon et al., 2022; Robertson et al., 2021; Touyz et al., 2020; Vall‐Roqué et al., 2021a; Weissman et al., 2020). According to a recent systematic review (Schneider et al., 2022), several studies show a negative influence of the pandemic on body image and disordered eating, but conversely, other studies report positive outcomes of the COVID‐19 pandemic, including reduction in eating disorder symptomatology, more time to reflect on recovery and engage in self‐care, greater motivation to recover, and more time to connect with family. These positive outcomes might be related with the socioeconomic status of participants, reflecting that those with higher social privilege have incurred fewer financial pressures, and this might have facilitated engagement with self‐care, recovery strategies, and social support (Schneider et al., 2022).

According to Rodgers et al. (2020), a pathway by which the current pandemic could increase eating disorders risk and symptoms is through an increased consumption of media (particularly social media) due to social distancing measures. This would happen through increased exposure to harmful eating and appearance‐related content, as well as more general stressful or traumatic world events. In this regard, in a recent study, it was found that there was a significant increase in the frequency of use of social network sites (SNS) and in the number of women following appearance‐focused accounts on Instagram during lockdown (Vall‐Roqué et al., 2021b). Taking into consideration that the use of social networks, especially an appearance‐focused use, is linked to body dissatisfaction (Fardouly & Vartanian, 2016; Holland & Tiggemann, 2016; Sherlock & Wagstaff, 2019), an increase in the (appearance‐focused) use of SNS during the pandemic could have led to an increase in body dissatisfaction. Furthermore, according to the tripartite influence model (Thompson et al., 1999), there are three primary influence variables that contribute to the development of body image disturbances: peers, parents, and media. In this sense, it has been stated that the tendency to engage in physical appearance comparisons plays a mediating role in the link between SNS use and body dissatisfaction (Fioravanti et al., 2022). Therefore, an increase in SNS use throughout the pandemic could be related to an increase in (media‐related) appearance comparisons. Also, considering that during the lockdown period people were presumably not engaging in socializing and could not compare themselves in‐person with their peers, they might have increased their comparisons on social media, and it is known that social media comparisons tend to be focused on the beauty ideal and can be particularly harmful (Fardouly et al., 2017), which might have led to increased body dissatisfaction. However, it could also be argued that, as during lockdown restrictions it was not allowed to engage in activities that often facilitate the appearance ideal social media self‐presentation, the appearance focused use of SNS might have been tempered.

It should be noted that the vast majority of research that has been published examining the impact of the pandemic on body image‐related variables or SNS use in community samples has used cross‐sectional designs, and there is limited research using experimental and longitudinal designs. The findings of the few studies that have longitudinally assessed the impact of the pandemic on body image are inconsistent: while there are studies that have found the pandemic to be associated with increased concerns about weight, shape and eating, and increased eating disorder symptomatology and screen time (e.g., Keel et al., 2020; Trott et al., 2021), others have found no differences between pre‐ and during/post‐lockdown in eating disorder symptoms and eating disorder risk (e.g., Koenig et al., 2021; Martínez‐de‐Quel et al., 2021). Regarding the studies that have longitudinally examined social media use over the pandemic, an increase in SNS use during the lockdown phase of the pandemic has been consistently reported (Marciano et al., 2022). For example, Fumagalli et al. (2021) indicated that social media use increased at the beginning of the lockdown (March–April 2020), and Arend et al. (2021) found that more than 40% of their participants increased their daily time spent using social media. However, to our knowledge, it has not been longitudinally assessed whether the pandemic has been associated with an appearance‐focused use of SNS.

Furthermore, to our knowledge, the possible changes in physical appearance comparisons throughout the pandemic have not been studied, and no studies have longitudinally assessed the (appearance‐focused) use of Instagram throughout the pandemic. Instagram is one of the most popular social media platforms worldwide, and recent research attention has turned specifically to this social network, as it is dedicated purely to the posting and sharing of photos, and it has been linked to body dissatisfaction (Fardouly & Vartanian, 2016; Horwitz, 2021; Tiggemann & Anderberg, 2020). When the first data collection of this study was conducted, Instagram was the most widely used photo‐based social network in Spain (IAB Spain, 2019, 2020). Finally, only a few studies have focused on women samples. Taking into consideration that body image problems are more prevalent among women than men (Myers & Crowther, 2009; O'Dea & Caputi, 2001) and considering that women have been reported to be an especially vulnerable group to the negative psychosocial effects of COVID‐19 (Ozamiz‐Etxebarria et al., 2020; Sun et al., 2020; Wang et al., 2020), special attention should be paid to this population group.

This is a four‐wave longitudinal study of data collection on Instagram use, body dissatisfaction and appearance comparisons in a Spanish women population. Participation was requested at four different times: the first one was some weeks before the pandemic outbreak, and the other ones took place approximately every 6 months throughout the pandemic. The objectives of this study were: (1) to determine the change in Instagram use, body dissatisfaction and physical appearance comparisons throughout the pandemic (both in the whole women sample and in two subgroups: those at risk of having an eating disorder and those without eating disorder risk), and (2) to explore whether there was a relationship between the changes in Instagram use throughout the pandemic and body dissatisfaction and physical appearance comparison tendency.

## 2 METHOD

### 2.1 Participants and procedure

A total of 272 Spanish women from a community sample completed questionnaire measures between November 2019 and January 2020 (period hereinafter referred to as T1 or baseline). These women were recruited in the context of a cross‐sectional study that aimed to validate the Spanish version of the Physical Appearance Comparison Scale‐Revised (PACS‐R; Vall‐Roqué et al., 2022) and to provide descriptive data on SNS use. The study included 1180 women aged between 16 and 70, but only 272 of them provided their email address and a code to be contacted later if necessary.

**FIGURE 1:**
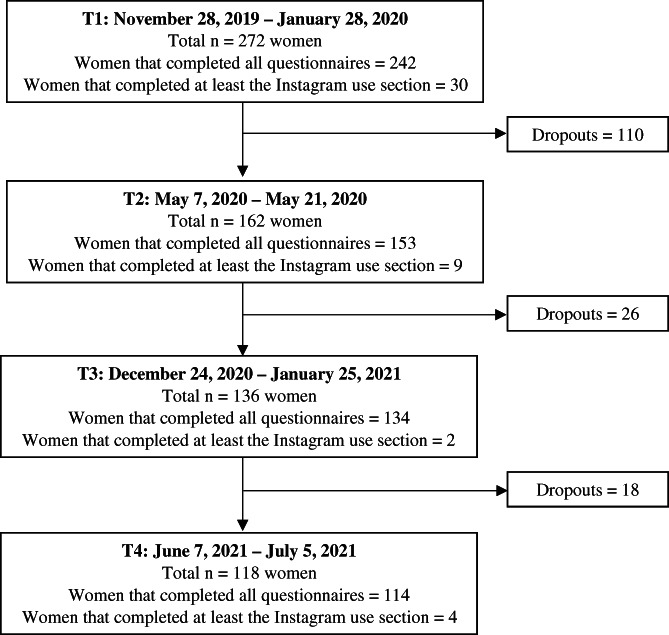
Flowchart of the participation in each data collection period

Women were contacted again in three different time periods throughout the pandemic (see Figure 1). In each time point, only women that had completed the questionnaire measures in the previous data collection period were contacted.

**FIGURE 2:**
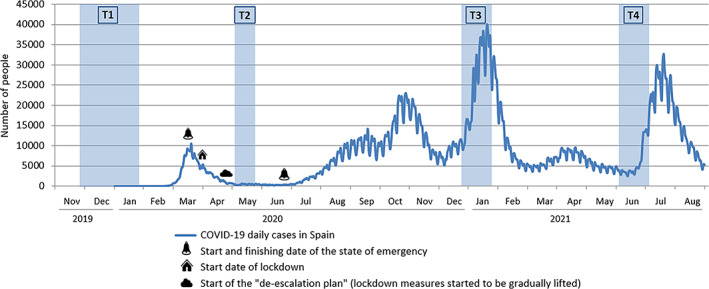
Time periods of data collection within the epidemic curve of the pandemic in Spain. Data extracted from https://cnecovid.isciii.es/covid19/#ccaa

Data collection periods, together with the epidemic curve of the pandemic in Spain, are graphically shown in Figure 2. It should be noted that the number of new daily infections was underestimated in Spain, especially during the first months of the pandemic (García‐García et al., 2021), therefore the number of COVID‐19 cases was higher than what is shown in the figure when the second data collection took place (T2).

At the beginning of the lockdown period, all Spanish population was mandated to remain in their residences except to go to work, purchase food and medicines, or attend emergencies. On March 28, 2020, the Spanish government suspended all nonessential activity as well, therefore all nonessential workers had to remain at home. Workers in some nonessential sectors who could not work remotely (e.g., industry and construction) were permitted to return to work on April 13, when the number of new cases and deaths in the country started to show a decreasing trend. On April 26, children under 14 were allowed to go out on short walks with their parents or other adults living in the same household. Exactly 2 days later, the Spanish government announced a four‐phase lockdown exit strategy for the country. This plan was divided into four phases (numbered 0–3), which were gradually implemented from May 2 until June 22, according to the epidemiological situation of each province. Phase 0 allowed Spanish population to engage in individual sport activities, including going for a walk, for 1 h a day. In Phase 1, individuals were allowed to visit friends and family in the same province, still with social distancing measures. Small businesses, religious sites, hotels, and terraces started to reopen under strict safety conditions. Phase 2 allowed theaters, museums, indoor restaurants, and bars to open with limited capacity. In Phase 3, all shops were back in business (with a maximum capacity of 50%), and meetings and conferences were allowed again (La Moncloa, 2020).

In the third and fourth data collection periods (T3 and T4), no complete lockdown was in place. However, in T3, the third wave of COVID‐19 was in full swing in Spain, and several restrictions were introduced: although these differed depending on the region of Spain, they included a night‐time curfew, a reduced maximum occupancy for restaurants, businesses and gatherings, and mobility restrictions. These restrictions caused great controversy because they coincided with New Year's festive season and therefore interfered with family gatherings and other celebrations. In T4, restrictions were relaxed but still included a maximum capacity for gatherings and limitations in the capacity and opening hours of retail, among others.

Inclusion criteria for participating in the initial study (which was conducted before the pandemic started) included living in Spain, being between 16 and 70 years old, and being a woman. Participants were recruited through various social media platforms (Twitter, Instagram, and Facebook) and through personal contacts of the research team. All measures were administered online through a secure internet‐based website. Participation was voluntary, and participants gave their informed consent before completing questionnaire measures. Participants did not receive compensation for their participation. Parental consent was not requested as the Spanish law states that it is only required for individuals under 14 years old for this type of studies (Organic Law 3/2018 for Data Protection and Guarantee of Digital Rights, articles 6 and 7). This study was performed in line with the principles of the Declaration of Helsinki. Approval was granted by the Bioethics Committee of the University of Barcelona (IRB00003099).

### 2.2 Measures

#### 2.2.1 Sociodemographic data

Information on age, gender, place of birth, place of residence, employment situation, average gross annual income, marital status, household members, height, and weight was systematically collected. Height and weight measures were used to calculate body mass index (BMI). Gender and educational level were only asked at T1.

#### 2.2.2 COVID‐19 and lockdown‐related information

In the second, third, and fourth waves, participants indicated whether themselves or a close person had contracted COVID‐19, and if they had a loved one that had deceased due to COVID‐19. This information was assessed using closed‐ended questions.

#### 2.2.3 Eating Disorders Inventory‐3 (Garner, 2004, adapted to Spanish by Elosua et al., 2010)

The Body Dissatisfaction subscale was used. This subscale consists of 10 items that assess discontentment with the overall shape and with the size of those regions of the body of extraordinary concern to those with eating disorders. Responses are rated on a 6‐point Likert scale, ranging from “Never” to “Always” (scores range: 0–40). The scale has an adequate internal consistency, with reported alpha values of .90 in Spanish women (Elosua et al., 2010). In this study, the alpha value for the Body Dissatisfaction subscale was .89 at all measurement waves.

#### 2.2.4 Physical Appearance Comparison Scale‐Revised (Schaefer & Thompson, 2014; adapted to Spanish by Vall‐Roqué et al., 2022)

The PACS‐R is an 11‐item questionnaire that measures the tendency to compare one's physical appearance to others in a broad array of social settings. Responses are rated on a 5‐point Likert‐type scale ranging from “Never” to “Always.” Higher scores indicate higher levels of general appearance comparison (scores range: 0–4). Psychometric evaluations of the Spanish version of the PACS‐R indicate that the questionnaire has a single factor structure, and excellent internal consistency (Cronbach's alpha of .97) and convergent validity in Spanish women (Vall‐Roqué et al., 2022). In this study, the alpha values for the PACS‐R were .97, .98, .97, and .97 at baseline, T2, T3, and T4, respectively.

#### 2.2.5 Eating Attitudes Test‐26 (Garner et al., 1982; adapted to Spanish by Castro et al., 1991)

The Eating Attitudes Test (EAT‐26) is a 26‐item self‐report questionnaire that measures disordered eating behavior and attitudes, and it is often used to identify individuals who might be at risk for an eating disorder. Items are presented in a 6‐point Likert scale ranging from “Never” to “Always,” and higher scores indicate higher levels of eating disturbances. It has three subscales: dieting, bulimia and food preoccupation, and oral control. The questionnaire has been reported to have adequate psychometric properties, and the alpha reliability coefficient in a Spanish sample was .93 (Castro et al., 1991). In this research, the alpha values for the EAT‐26 were .93, .92, .93, and .94 at baseline, T2, T3, and T4, respectively.

The EAT‐26 was only used to divide the sample between those at risk of having an eating disorder (total score ≥ 20) and those without eating disorder risk (total score < 20).

#### 2.2.6 Instagram use

A Likert‐scale self‐report item assessed the frequency of Instagram use in each time point (*I do not have an Instagram account*, *Never/Almost never*, *Less than 1 h/day*, *1–2 h/day*, *2–3 h/day*, *3–4 h/day*, *More than 4 h/day*).

A multiple answer question queried which type of accounts individuals followed on Instagram (only applicable to those participants who reported having an Instagram account) in each wave of data collection. Participants who indicated that they followed fashion, clothing brands, weight‐loss tips or diets, beauty, or fitness accounts were included in the “following appearance‐focused accounts” group, whereas those who did not follow any of the mentioned accounts were included in the “not following appearance‐focused accounts” group.

Moreover, three groups of participants were created according to whether their frequency of Instagram use had increased, remained the same or decreased during the pandemic (from T1 to T2, from T1 to T3, and from T1 to T4). Similarly, participants were categorized into three different groups depending on whether they had started following appearance‐focused accounts on Instagram during the pandemic, they had stopped following appearance‐focused accounts, or they had continued following or not following appearance‐focused accounts.

### 2.3 Data analysis

Data was analyzed using SPSS version 25 and STATA 15.0 software.

Descriptive statistics (frequency and percentages for categorical variables, mean and standard deviation [*SD*] for continuous variables) were used for assessing the sociodemographic and COVID‐19‐related characteristics of the sample, the Instagram use variables, and the psychological variables. Cochran's Q tests, Friedman tests, marginal homogeneity tests, and repeated‐measures analysis of variances (ANOVAs) were conducted to explore whether there were significant differences in the sociodemographic and COVID‐19 variables among the four data collection waves. Cochran's Q tests were used to determine if there were differences on dichotomous variables between three or more related groups. Friedman tests were used to examine if there were differences between groups when the variable being measured was ordinal. Marginal homogeneity tests were used to determine if there were differences on categorical nondichotomous variables between related groups.

For the categorical variables “frequency of Instagram use” and “following appearance‐focused Instagram accounts,” the Cochran's Q test was used to explore whether there were significant differences among the four time points. Significant results were followed by Bonferroni adjustment.

Student's *t* tests were conducted to explore whether there were significant differences in body dissatisfaction and physical appearance comparisons at baseline between individuals with and without eating disorder risk. To assess the differences among time points in the psychological variables, mixed regression models with repeated measures were used, which included the psychological variables (body dissatisfaction and physical appearance comparisons) as dependent variables, adjusting for the interaction among risk of eating disorders (dichotomous variable), time as a repeated measure, and its main effects. Baseline measures (T1) were selected as the reference category. BMI and educational level were included as covariates in the model, as a previous bivariate analysis was performed between the sociodemographic/COVID‐19‐related characteristics and the outcome variables, and the bivariate associations with demonstrated levels of statistical significance (*p* < .05) were included in the mixed regression models. In the models with significant or marginally significant interaction, mixed regression models were performed for each of the interaction subgroups. Participant and intercept were introduced as random factors in all models. The intraclass correlation was obtained for each model. Time was treated as a categorical variable to allow the model to fit every possible pattern in time and not assume linear growth, considering that a disadvantage that has been reported of modeling time as a continuous variable is that the development over time of the outcome variable *Y* is modeled as a linear function (Twisk, 2013).

One‐way ANOVAs were carried out to assess whether the changes in the frequency of Instagram use (increase, decrease, or no changes) and the changes in following appearance‐focused accounts on Instagram throughout the pandemic were significantly associated with body dissatisfaction and appearance comparison tendency. If Levene's test of homogeneity of variance was violated, the Brown–Forsyth statistic was used.

Hedges' *g* (Hedges & Olkin, 1985) was calculated after carrying out Student's *t*‐tests and ANOVAs to measure the effect sizes of statistically significant results. Cohen's criteria (Cohen, 1988) were used to interpret effect sizes, where .2, .5, and .8 represented small, medium, and large effects, respectively.

Missing values were treated by means of multiple imputation procedures (White et al., 2011) with results based on 100 imputed datasets (missing values from the follow‐up measurements were imputed). More details on the imputation process can be found in the Supporting Information.

## 3 RESULTS

### 3.1 Descriptive characteristics of the sample

Participants ranged in age from 16 to 70, with a mean age of 34.43 years (*SD* = 11.07) at baseline. Almost 70% of the sample had tertiary level education, and the mean BMI at baseline was 23.45 (*SD* = 4.39). Descriptive statistics of sociodemographic and COVID‐19 related variables are reported in Table 1. Statistically significant differences were found among measurement waves in the following variables: *age* (*F* (3115) = 50,148.52, *p* < .001 among all time points, being higher in each time point; Hedges' *g* [95% CI] ranged from .07 [−.12–.27] for T1–T2, to .24 [.02–.46] for T1–T4), *BMI* (*F* (3112) = 2.85, *p* = .037, significant increase from T1 to T4, Hedges' *g* [95% CI] = .11 [.11–.33]), *employment status* (significant increase from T2 to T4 in the proportion of participants employed [Cochran's Q, *χ* ^2^ (3) = 9.23, *p* = .026], and significant decrease from T2 to T4 in the proportion of participants unemployed [Cochran's Q, *χ* ^2^ (3) = 9.78, *p* = .021]), *household members (“living with”)* (significant differences between T1 and T3 and between T2 and T3: marginal homogeneity test, *χ* ^2^ = 2.46, *p* = .014 for T1–T3; *χ* ^2^ = 2.39, *p* = .017 for T2–T3; in T3, the proportion of participants living with their partner increased compared to T1 or T2, and the proportion of participants living with flatmates decreased).

**TABLE 1:** Descriptive characteristics of participants' sociodemographic data and of coronavirus disease‐2019‐related variables

	Baseline (*n* = 272)	T2 (*n* = 162)	T3 (*n* = 136)	T4 (*n* = 118)
Age (years), mean (*SD*)	34.43 (11.07)	35.22 (11.53)	36.07 (11.32)	37.13 (11.43)
BMI (kg/m^2^), mean (*SD*)	23.45 (4.39)	23.89 (4.86)	23.64 (4.46)	23.95 (4.9)
Educational level, *n* (%)				
Primary education	11 (4.1)	–	–	–
Secondary education	73 (26.8)	–	–	–
Tertiary education	188 (69.2)	–	–	–
Employment status^a^, *n* (%)				
Not working	27 (9.9)	12 (7.5)	11 (8.1)	9 (7.6)
Student	77 (28.3)	48 (29.6)	31 (22.8)	25 (21.2)
Employee	139 (51.1)	74 (45.7)	75 (55.1)	73 (61.9)
Self‐employed	37 (13.6)	21 (13.0)	17 (12.5)	16 (13.6)
Temporary leave	4 (1.5)	3 (1.9)	6 (4.4)	5 (4.2)
Unemployed	27 (9.9)	25 (15.4)	15 (11.0)	10 (8.5)
Retired	6 (2.2)	4 (2.5)	3 (2.2)	3 (2.5)
Average gross annual income, *n* (%)				
No income	61 (22.4)	34 (21)	22 (16.2)	16 (13.6)
<1 MW	43 (15.8)	27 (16.7)	27 (19.9)	19 (16.1)
1–2 MW	65 (23.9)	35 (21.6)	30 (22.1)	31 (26.3)
2–3 MW	44 (16.2)	35 (21.6)	24 (17.6)	22 (18.6)
3–4 MW	34 (12.5)	17 (10.5)	24 (17.6)	18 (15.3)
4–5 MW	17 (6.3)	11 (6.8)	6 (4.4)	9 (7.6)
>5 MW	8 (2.9)	3 (1.9)	3 (2.2)	3 (2.5)
Marital status, *n* (%)				
Single	67 (24.6)	33 (20.4)	27 (19.9)	22 (18.6)
In a relationship/married	186 (68.4)	119 (73.5)	98 (72)	87 (73.7)
Divorced/widowed	19 (7)	10 (6.1)	11 (8)	9 (7.6)
Living with (household members), *n* (%)				
Alone	18 (6.6)	7 (4.3)	8 (5.9)	8 (6.8)
With partner	111 (40.8)	66 (40.7)	63 (46.3)	52 (44.1)
With family member(s)	101 (37.1)	72 (44.4)	53 (39)	48 (40.7)
With flatmate(s)	40 (14.7)	16 (9.8)	11 (8.1)	10 (8.5)
Other	2 (0.7)	1 (0.6)	1 (0.7)	–
*COVID‐19 related variables*				
Contracted COVID‐19, *n* (%)				
Yes		1 (0.6)	9 (6.6)	17 (14.4)
No		118 (72.8)	102 (75)	87 (73.7)
Not sure		43 (26.5)	25 (18.4)	14 (11.9)
Loved one infected with COVID‐19, *n* (%)				
Yes		63 (38.9)	95 (69.9)	89 (75.4)
No		78 (48.1)	34 (25)	25 (21.2)
Not sure		21 (13)	7 (5.1)	4 (3.4)
Loved one deceased due to COVID‐19, *n* (%)				
Yes		18 (11.1)	29 (21.3)	32 (27.1)
No		144 (88.9)	107 (78.7)	86 (72.9)

Abbreviations: MW, minimum wage in Spain in 2019; *SD*, standard deviation; T, time.

^a^ Selecting more than one answer was possible.

Statistically significant differences were found among measurement waves in all COVID‐19 related variables: *COVID‐19 contraction* (the proportion of participants that contracted the disease significantly increased in each follow‐up after T2; marginal homogeneity test, *χ* ^2^ = 3.43, *p* = .001 for T2–T3; *χ* ^2^ = 4.90, *p* < .001 for T2–T4; *χ* ^2^ = 2.61, *p* = .009 for T3–T4); *loved one infected with COVID‐19* (the proportion of participants that had a close person who had had the disease significantly increased in each follow‐up after T2; marginal homogeneity test, *χ* ^2^ = 6.00, *p* < .001 for T2–T3; *χ* ^2^ = 6.38, *p* < .001 for T2–T4; *χ* ^2^ = 2.67, *p* = .008 for T3–T4); *loved one deceased due to COVID‐19* (the proportion of participants that had a close person who deceased due to COVID‐19 significantly increased in each follow‐up after T2; Cochran's Q test, *χ* ^2^ (2) = 29.79, *p <* .001, significant differences between T2–T3 and T2–T4).

### 3.2 Instagram use

Table 2 summarizes the frequency of Instagram use and whether participants followed Instagram appearance‐focused accounts in each time point. No statistically significant changes were found in the frequency of Instagram use among the different data collection waves (Friedman's test, *χ* ^2^ (3) = 6.09, *p* = .107). Also, there was not a statistically significant change (although there was a trend toward significance) on the number of people following appearance‐focused accounts on Instagram among the different waves (Cochran's Q test, *χ* ^2^ (3) = 6.51, *p* = .089).

**TABLE 2:** Frequency of Instagram use and frequency of participants following Instagram appearance‐focused accounts in each data collection period

	Baseline	T2	T3	T4
Frequency of Instagram use, *n* (%)				
No Instagram account	44 (16.2)	38 (14)	29 (10.5)	23 (8.3)
Never/almost never uses Instagram	30 (11)	32 (11.9)	42 (15.3)	30 (11)
Less than half an hour/day	46 (16.9)	51 (18.7)	43 (15.9)	47 (17.4)
Half an hour to 1 h/day	51 (18.8)	52 (19)	57 (21.1)	77 (28.2)
1–2 h/day	60 (22.1)	50 (18.4)	64 (23.6)	49 (18.1)
2–3 h/day	24 (8.8)	29 (10.5)	25 (9.3)	23 (8.5)
3–4 h/day	12 (4.4)	11 (4.1)	12 (4.3)	13 (4.6)
More than 4 h/day	5 (1.8)	9 (3.4)	0 (0)	10 (3.8)
Following appearance‐focused accounts, *n* (%)				
Yes	87 (32)	91 (33.4)	99 (36.4)	101 (37.1)
No	185 (68)	181 (66.6)	173 (63.6)	171 (62.9)

Abbreviation: T, time.

### 3.3 Body dissatisfaction and physical appearance comparisons

Body dissatisfaction and physical appearance comparison scores are presented in Table 3 for each time point, both for the whole sample and for individuals with and without risk of having an eating disorder (according to their EAT‐26 score). The mean baseline scores of body dissatisfaction and physical appearance comparisons for those at risk of having an eating disorder were significantly higher (with high effect sizes) than the scores of those without eating disorder risk (body dissatisfaction: *t* = −9.84, *p* < .001; Hedges' *g* [95% CI] = 1.63 [1.29–1.97]; physical appearance comparisons: *t* = −10.25, *p* < .001; Hedges' *g* [95% CI] = 1.74 [1.40–2.09]).

**TABLE 3:** Scores in body dissatisfaction and physical appearance comparison in each measurement wave (for the whole sample and divided by eating disorder risk)

	Baseline	T2	T3	T4
Body dissatisfaction, mean (*SD*)				
Whole sample (*n* = 272)	14.12 (9.74)	14.65 (9.75)	14.50 (9.62)	15.30 (9.49)
Sample with ED risk (*n* = 47)	25.33 (8.53)	24.86 (9.40)	23.50 (9.95)	23.83 (9.88)
Sample without ED risk (*n* = 225)	11.78 (8.24)	12.53 (8.37)	12.62 (8.40)	13.53 (8.36)
Physical appearance comparison, mean (*SD*)				
Whole sample (*n* = 272)	1.60 (1.18)	1.82 (1.20)	1.73 (1.17)	1.79 (1.19)
Sample with ED risk (*n* = 47)	3.03 (1.05)	3.16 (0.91)	2.92 (1.03)	2.94 (1.08)
Sample without ED risk (*n* = 225)	1.31 (0.97)	1.54 (1.05)	1.48 (1.03)	1.55 (1.06)

Abbreviations: ED, eating disorder; *SD*, standard deviation; T, time.

To assess the differences in body dissatisfaction and physical appearance comparisons among the different time points, mixed regression models with repeated measures were used. In the regression model for body dissatisfaction (Table 4), body dissatisfaction significantly increased from T1 to T4 (*Β* = 1.76; *p* < .001). The interaction between time and eating disorder risk was statistically significant at T3 (*p* = .028) and T4 (*p* = .017). BMI was significantly associated with changes in body dissatisfaction (*p* < .001): body dissatisfaction score increased .79 for each BMI unit. The educational level was not found to significantly predict body dissatisfaction.

**TABLE 4:** Regression model for body dissatisfaction and physical appearance comparison tendency

	Body dissatisfaction						Physical appearance comparison tendency					
	Whole sample		Sample with ED risk		Sample without ED risk		Whole sample		Sample with ED risk		Sample without ED risk	
	*B* (Std. Err.)	*p*	*B* (Std. Err.)	*p*	*B* (Std. Err.)	*p*	*B* (Std. Err.)	*p*	*B* (Std. Err.)	*p*	*B* (Std. Err.)	*p*
T2 (ref: T1)	.74 (.44)	.089	−.48 (0.99)	.633	.74 (.44)	.089	.23 (.06)	**<.001**	.14 (.13)	.313	.23 (.06)	**<.001**
T3 (ref: T1)	.84 (.47)	.075	−1.83 (1.13)	.107	.84 (.47)	.075	.17 (.07)	**.009**	−.11 (.15)	.483	.17 (.17)	**.009**
T4 (ref: T1)	1.75 (.48)	**<.001**	−1.50 (1.29)	.247	1.75 (.48)	**<.001**	.24 (.07)	**.001**	−.09 (.15)	.576	.24 (.24)	**<.001**
EAT (Risk of ED) (ref: No Risk of ED)	13.00 (1.32)	**<.001**					1.72 (.18)	**<.001**				
Time × EAT (ref: T1 − No risk of ED)												
T2 − Risk of ED	−1.22 (1.06)	.251					−.09 (.15)	.534				
T3 − Risk of ED	−2.67 (1.21)	**.028**					−.28 (.17)	.093				
T4 − Risk of ED	−3.24 (1.35)	**.017**					−.33 (.127)	.053				
BMI	.79 (.10)	**<.001**	.68 (.23)	**.004**	.83 (.12)	**<.001**	.01 (.01)	.506	−.003 (.03)	.927	.01 (.02)	.399
Educational level: Tertiary education (ref: Primary or secondary education)	−.81 (1.08)	.457	−2.40 (2.60)	.355	−.32 (1.17)	.783	.02 (.14)	.866	−.001 (.29)	.997	.04 (.15)	.814
Constant	−6.01 (2.61)	.021	10.24 (5.75)	.075	−7.40 (2.95)	.012	1.08 (.35)	.002	3.09 (.68)	<.001	.97 (.40)	.015
	*F* (9, 7266.1) = 14.74; *p* < .001; ICC = .752		*F* (5, 3702.3) = 2.11; *p* = .062; ICC = .797		*F* (5, 6053.2) = 11.67; *p* <. 001; ICC = .738		*F* (9, 8846.4) = 11.08; *p* < .001; ICC = .705		*F* (5, 5503.6) = .78; *p* = .567; ICC = .706		*F* (5, 5244.8) = 3.85; *p* = .002; ICC = .704	

*Note*: The dependent variables of the model were Body dissatisfaction and Physical appearance comparison tendency.

Bold values represent *p*‐values < .05

Abbreviations: BMI, body mass index; EAT, Eating attitudes test‐26; ED, eating disorder; ICC, intraclass correlation coefficient; T, time.

In the subgroups analysis, it was found that the sample that was not at risk of having an eating disorder significantly increased their body dissatisfaction at T4 (*Β* = 1.75; *p* < .001) (with respect to T1), and with a trend toward significance at T2 (*p* = .089) and T3 (*p* = .075). However, no significant changes in body dissatisfaction among time points were found in the sample at risk of having an eating disorder. In both subgroups, BMI was found to be a statistically significant predictor of body dissatisfaction.

Regarding physical appearance comparisons (Table 4), they significantly increased from T1 to T2 (*B* = .23; *p* < .001), T3 (*B* = .17; *p* = .009), and T4 (*Β* = .24; *p* = .001). The interaction between time and eating disorder risk showed a trend toward significance at T3 (*p* = .093) and T4 (*p* = .053). The effect of eating disorder risk was significant, with a score in appearance comparisons 1.72 times higher in women with eating disorder risk versus those without eating disorder risk (*p* < .001).

In the subgroup of women without eating disorder risk, physical appearance comparisons significantly increased at T2 (*Β* = .23; *p* < .001), T3 (*Β* = .17; *p* = .009), and T4 (*Β* = .24; *p* < .001) with respect to T1. In contrast, no significant differences were found in appearance comparisons among time points in the group of women with risk of having an eating disorder.

### 3.4 Relationship between the changes in Instagram use throughout the pandemic and body dissatisfaction and physical appearance comparisons

As shown in Table 5, no significant differences were found in body dissatisfaction and physical appearance comparisons at T4 depending on whether participants' Instagram frequency of use had increased, remained the same or decreased from T1 to T4. The same pattern was observed when the differences between T1–T2 and T1–T3 were assessed.

**TABLE 5:** Differences in body dissatisfaction and physical appearance comparisons according to participants' changes in Instagram use

	T1–T2			T1–T3			T1–T4		
	%	Body dissatisfaction (T2)	Physical appearance comparisons (T2)	%	Body dissatisfaction (T3)	Physical appearance comparisons (T3)	%	Body dissatisfaction (T4)	Physical appearance comparisons (T4)
Changes in the frequency of Instagram use									
Increase in use	25.3	*F* (2, 219) = .92 *p* = .401	*F* (2, 221) = .56 *p* = .573	27.7	*F* (2, 220.7) = 1.01 *p* = .365	*F* (2, 234.2) = 1.68 *p* = .189	39	*F* (2, 207.3) = .47 *p* = .624	*F* (2, 225.8) = .57 *p* = .567
Decrease in use	19.8			23.4			25.4		
No changes in use	54.9			48.9			35.6		
Changes in following appearance‐focused accounts on Instagram									
Started following appearance‐focused accounts	11.1	*F* (2, 238.4) = .61 *p* = .542	*F* (2, 232.8) = .59 *p* = .556	15.4	*F* (2, 212.9) = 0.19 *p* = .824	*F* (2, 218) = 0.24 *p* = .784	14.4	*F* (2, 200) = 1.14 *p* = .321	*F* (2, 210.5) = 0.60 *p* = .552
Stopped following appearance‐focused accounts	8.6			7.4			6.8		
Continued following or not following appearance‐focused accounts	80.2			77.2			78.8		

Similarly, no significant differences were found in body dissatisfaction and physical appearance comparisons at T4 depending on whether participants had started following appearance‐focused accounts on Instagram during the pandemic, had stopped following appearance‐focused accounts, or had continued following or not following appearance‐centered Instagram accounts throughout the different data collection periods. The same was observed when the differences between T1–T2 and T1–T3 were assessed (Table 5).

## 4 DISCUSSION

The general aim of this study was to explore the changes in body dissatisfaction, physical appearance comparisons, and Instagram use throughout the pandemic in a community sample of Spanish women, and to determine whether there was an association between the changes in Instagram use throughout the pandemic and body dissatisfaction and physical appearance comparisons.

Regarding the characteristics of this study's sample, we found that women's BMI increased throughout the pandemic, although the effect size of this significant increase was very small and should therefore be interpreted with caution. The literature is inconsistent in this regard: while some studies have reported an increase in BMI throughout the pandemic, others have found no changes or a decrease in women's BMI (Bakaloudi et al., 2021). BMI and weight trajectories during COVID‐19 are probably dependent on many factors that should be considered, such as age, socioeconomic status, pandemic living and working conditions, diet, physical activity, or alcohol intake (Khan et al., 2022). Moreover, according to our regression model, a higher BMI was found to be associated with increased body dissatisfaction. The association between a higher BMI and body dissatisfaction has been consistently reported in other studies (e.g., Quittkat et al., 2019).

Moreover, with regards to the COVID‐19‐related variables, the percentage of women that had suffered COVID‐19 or that had a close person who had contracted the virus or deceased due to COVID‐19 significantly increased in each data collection wave. This reflects how COVID‐19 was more present to everybody's lives as the pandemic progressed.

Our findings suggest that there were not significant changes in the frequency of Instagram use throughout the pandemic. Similarly, there were not significant changes in the proportion of women following appearance‐focused accounts on Instagram among the different data collection time periods. These results do not align with the scarce published literature (Fernandes et al., 2020; Vall‐Roqué et al., 2021b), and this might be partly due to the longitudinal design used in this research, which included pre‐pandemic data, versus the cross‐sectional designs relying on retrospective reports used in the other studies. However, it should be considered that, although our results did not yield statistical significance, the percentage of women that did not have an Instagram account decreased from 16% to 8% from baseline (before the pandemic outbreak) to the last data collection period (almost a year and a half after the start of the pandemic), and the percentage of women who used Instagram more than 4 h per day raised from 1.8% to 3.8%. Similarly, in each data collection period, the percentage of women that followed appearance‐focused accounts on Instagram increased. Therefore, it is possible that the pandemic is associated with changes in Instagram use, but these changes might be more complex (and less evident) than they seem in cross‐sectional studies.

The pandemic seems to have had an effect on body dissatisfaction and physical appearance comparisons. Regarding body dissatisfaction, although its increase was not linear throughout the pandemic, there was a significant raise in body dissatisfaction from baseline to our last data collection period. This aligns with most of the published literature, which states the negative impacts of the COVID‐19 pandemic on appearance, shape, and weight concerns (Schneider et al., 2022). Similarly, there was a significant increase in physical appearance comparisons from baseline to the different data collection periods. To our knowledge, this is the first study to longitudinally assess the changes on appearance comparisons throughout the pandemic. Considering that the tendency to engage in appearance comparisons has been reported to be related to body dissatisfaction and eating disturbances (Thompson et al., 1999), this increase in appearance comparisons throughout the pandemic, together with the increase in body dissatisfaction, should be paid special attention as it might lead to subsequent eating disturbances or eating disorders. Moreover, considering the social distancing measures that have been in place, it is possible that current appearance comparisons occur to a greater extent in an online context (i.e., SNS, where the thin and beauty ideal is often displayed, and users easily portray a rosy image of their lives) rather than offline. Future research could shed light on this.

The findings of this study indicate that the increase in body dissatisfaction and appearance comparisons throughout the pandemic was only significant in those individuals that were not at risk of having an eating disorder at baseline, while those with eating disorder risk did not show significant changes in their body satisfaction or appearance comparisons. Although these results need to be considered carefully as the sample with eating disorder risk was small, they suggest that women's general population's body image has worsened throughout the pandemic, but this is not the case for those with previous psychopathology, therefore we could hypothesize that those with eating disturbances were already experiencing body dissatisfaction and high appearance comparisons before the pandemic, and their symptoms did not worsen (nor improve) throughout the pandemic. Even though the vast majority of the literature suggests a negative impact of the pandemic on people with eating disorders, as indicated by Schneider et al. (2022) in their systematic review, some studies have reported positive aspects of COVID‐19, including reduction in eating disorder symptomatology and greater motivation to recover from an eating disorder. Hence, the effects of the pandemic on people living with an eating disorder might be complex and need further research. Regarding women without previous eating disorder risk, further work is needed to understand mechanisms underpinning the changes in body dissatisfaction and appearance comparisons in the general population. According to Robertson et al. (2021) and Schneider et al. (2022), potential explanations or factors associated with changes in body image disturbances could include higher levels of stress, worry and anxiety (for example, resulting from increased caregiving responsibilities), increased levels of rumination, loneliness and depression, fear of COVID‐19, exposure to increased weight stigma via public health and social media messaging regarding obesity and COVID‐19, increased saliency of food and eating as a result of shopping restrictions and changes to daily routines, household arguments or family conflicts, and changes in living situation and access to usual support networks. Another potential explanation could be related to the fact that, as the population was suddenly mandated to remain at home for longer periods of time, individuals replaced their “unfiltered” in‐person comparisons with online comparisons with influencers that promoted the beauty ideal. These changes in body dissatisfaction and appearance comparisons in community women highlight the importance of implementing body image and eating disorders prevention programs and initiatives (e.g., media literacy programs).

However, an increase or decrease in Instagram use throughout the pandemic, or the fact of starting to follow or unfollow appearance‐focused accounts on Instagram, was not associated with higher or lower levels of body dissatisfaction or appearance comparisons. This suggests that the changes in Instagram use during the pandemic might not be necessarily associated with body image problems. Staniewski and Awruk (2022) recently reported in their study a positive impact of Instagram on mental well‐being during the pandemic, while other authors have stated that social media exposure during the pandemic may predict disordered eating symptoms (Bellapigna et al., 2021). These contradictory findings might be due to several reasons, including the different measures used in each study and the potential impact of confounding variables. Also, some authors have suggested that the specific social media activities (e.g., greater investment in “selfie” activities), rather than the total time spent using it, predicts body dissatisfaction (Cohen et al., 2018). In this line, as mentioned before, appearance comparisons on SNS (instead of changes in the frequency of use or in the types of accounts followed) might better explain increased appearance comparisons and body dissatisfaction. Hence, future research could focus on the effect of specific online activities performed during the pandemic and their impact on body image‐related variables.

## 5 LIMITATIONS AND FUTURE DIRECTIONS

To our knowledge, this is the first study that longitudinally assesses the changes in body dissatisfaction, physical appearance comparisons, and Instagram use throughout the COVID‐19 pandemic using pre‐pandemic data. Its limitations lead to several future directions. First, the sample size of this study was small, and there was a high attrition rate. Second, this study included a community sample and, although there were some women at risk of having an eating disorder, future research should examine samples with eating disorders. Third, although women are more susceptible to appearance comparisons and body dissatisfaction (Myers & Crowther, 2009), future studies should also study men and individuals who do not identify within the gender binary system. Fourth, other SNS should be assessed, such as TikTok, which is growing rapidly and has been associated with eating disorders‐related content during the pandemic (Lladó Jordan et al., 2021). Fifth, we employed a survey measurement to assess participants' perceived Instagram use, which might be less accurate than in‐situ measurements (Naab et al., 2018). Future studies could use more objective digital trace data sources to measure SNS use. Sixth, the measure that we used to assess physical appearance comparisons poses two potential limitations: (1) although it examines comparisons in several social contexts, it does not specifically assess comparisons on social media; future research could include specific items about comparisons on social media, and (2) some items of this questionnaire refer to social situations that were prohibited during the strict phase of lockdown (e.g., eating in a restaurant or going shopping). Although the scale asks respondents to answer items thinking in what they usually do (and not in the specific moment of answering the questionnaire), this should be taken into consideration. Finally, as participants' recruitment was conducted especially online, the sample of this study is not representative of the Spanish population.

## CONSENT TO PARTICIPATE

Informed consent was obtained from all participants included in the study. Parental consent was not requested as the Spanish law (Organic Law 3/2018) states that it is not required for individuals who are 14 years old or older for this type of studies.

## AUTHOR CONTRIBUTIONS

**Helena Vall‐Roqué:** Conceptualization; data curation; formal analysis; investigation; methodology; visualization; writing – original draft; writing – review and editing. **Ana Andrés:** Conceptualization; formal analysis; methodology; supervision; writing – review and editing. **Himar González‐Pacheco:** Formal analysis; methodology; supervision; writing – review and editing. **Carmina Saldaña:** Conceptualization; project administration; resources; software; supervision; writing – review and editing.

## FUNDING INFORMATION

This research did not receive any specific grant from funding agencies.

## CONFLICT OF INTEREST

None.

## Supporting information


**Appendix S1.** Supporting Information

## Data Availability

The datasets generated and/or analyzed during the current study are available from the corresponding author on reasonable request.
