# Supplementary material for: Women's body dissatisfaction, physical appearance comparisons, and Instagram use throughout the COVID‐19 pandemic: A longitudinal study
Source: Int J Eat Disord. 2022 Oct 21;56(1):118–31. doi: 10.1002/eat.23827 (PMC10092427; doi:10.1002/eat.23827)
Supplement: Supplementary file 1 — Appendix S1. Supporting Information [file EAT-56-118-s001.docx]

**SUPPORTING INFORMATION FILE**

**Multiple imputation model**

Multiple imputation was performed by means of mi impute chained using the software Stata 15.0. The following variables were considered regular and used as predictors to perform imputations: age, employment status and average gross annual income. A total of 21 variables were imputed. Imputation of variables was organized starting from those that had less data lost (e.g., age). Each variable was imputed in chronological order: T1 first and afterwards T2, T3 and T4. As a general rule, the latest available information of the variable to impute was used. When information from other variables was used, the information from the same time moment was used. A total of 100 imputations were performed for every missed data. Due to convergence problems, not all the variables collected could be included.

The following table shows the order of imputation of the variables, the variables used in the imputation, the prediction model, and the number of lost data for this variable.

|  | **Imputed variable** | **Variables used in the imputation** | **Imputation Model** | **N missed** |
| --- | --- | --- | --- | --- |
| 1 | Age (years) | Educational level, Average gross annual income | Pmm, knn(3) | 2 |
| 2 | BMI T1 | Educational level, Average gross annual income, Age | Pmm, knn(3) | 2 |
| 3 | BMI T2 | Educational level, Average gross annual income, Age | Pmm, knn(3) | 111 |
| 4 | BMI T3 | Educational level, Average gross annual income, Age | Pmm, knn(3) | 135 |
| 5 | BMI T4 | Educational level, Average gross annual income, Age | Pmm, knn(3) | 154 |
| 6 | Body dissatisfaction T1 | Educational level, Average gross annual income, BMI T1 | Pmm, knn(3) | 13 |
| 7 | Body dissatisfaction T2 | Educational level, Average gross annual income, Age, BMI T2, Body dissatisfaction T1 | Pmm, knn(3) | 116 |
| 8 | Body dissatisfaction T3 | Educational level, Average gross annual income, Age, BMI T3, Body dissatisfaction T2 | Pmm, knn(3) | 136 |
| 9 | Body dissatisfaction T4 | Educational level, Average gross annual income, Age, BMI T4, Body dissatisfaction T3 | Pmm, knn(3) | 158 |
| 10 | Eating disorder risk | Educational level, Average gross annual income, Age, BMI T4, Body dissatisfaction T1 | Pmm, knn(3) | 19 |
| 11 | Physical appearance comparison tendency T1 | Educational level, Average gross annual income, Age, BMI T1, Body dissatisfaction T1 | Pmm, knn(3) | 30 |
| 12 | Physical appearance comparison tendency T2 | Educational level, Average gross annual income, Age, BMI T2, Body dissatisfaction T2, Physical appearance comparison tendency T1 | Pmm, knn(3) | 119 |
| 13 | Physical appearance comparison tendency T3 | Educational level, Average gross annual income, Age, BMI T3, Body dissatisfaction T3, Physical appearance comparison tendency T2 | Pmm, knn(3) | 138 |
| 14 | Physical appearance comparison tendency T4 | Educational level, Average gross annual income, Age, BMI T4, Body dissatisfaction T4, Physical appearance comparison tendency T3 | Pmm, knn(3) | 158 |
| 15 | Frequency of Instagram use T1 | Educational level, Average gross annual income, Age, BMI T1, Body dissatisfaction T1 | mlogit | 44 |
| 16 | Frequency of Instagram use T2 | Educational level, Average gross annual income, Age, BMI T2, Body dissatisfaction T2, Frequency of Instagram use T1 | mlogit | 132 |
| 17 | Frequency of Instagram use T3 | Educational level, Average gross annual income, Age, BMI T3, Body dissatisfaction T3, Frequency of Instagram use T2 | mlogit | 149 |
| 18 | Frequency of Instagram use T4 | Educational level, Average gross annual income, Age, BMI T4, Body dissatisfaction T4, Frequency of Instagram use T3 | mlogit | 162 |
| 19 | Following appearance-focused accounts T2 | Educational level, Average gross annual income, Age, BMI T2, Body dissatisfaction T2, Frequency of Instagram use T2, Following appearance-focused accounts T1 | logit | 110 |
| 20 | Following appearance-focused accounts T3 | Educational level, Average gross annual income, Age, BMI T3, Body dissatisfaction T3, Frequency of Instagram use T3, Following appearance-focused accounts T2 | logit | 136 |
| 21 | Following appearance-focused accounts T4 | Educational level, Average gross annual income, Age, BMI T4, Body dissatisfaction T4, Frequency of Instagram use T4, Following appearance-focused accounts T3 | logit | 154 |
